# Clinical Presentation, Outcomes, and Treatment of Membranous Nephropathy after Transplantation

**DOI:** 10.1155/2018/3720591

**Published:** 2018-07-05

**Authors:** Artur Q. B. da Silva, Taina V. de Sandes-Freitas, Juliana B. Mansur, Jose Osmar Medicina-Pestana, Gianna Mastroianni-Kirsztajn

**Affiliations:** ^1^Federal University of São Paulo (UNIFESP), São Paulo, SP, Brazil; ^2^Federal University of Ceará (UFC), Fortaleza, CE, Brazil

## Abstract

There are scarce data about clinical presentation and outcomes of posttransplant membranous nephropathy (MN), and few reports include a large number of patients. This was a retrospective cohort including adult patients with posttransplant MN transplanted between 1983 and 2015 in a single center (n=41). Only patients with histological diagnosis of MN in kidney grafts were included. Clinical and laboratory presentation, histological findings, treatment, and outcomes were detailed. Patients were predominantly male (58.5%), with a mean age of 49.4 ± 13.2 years; 15 were considered as recurrent primary MN; 3 were class V lupus nephritis; 14 were considered as* de novo* cases, 7 secondary and 7 primary MN; and 9 cases were considered primary but it was not possible to distinguish between* de novo* MN and recurrence. Main clinical presentations were proteinuria (75.6%) and graft dysfunction (34.1%). Most patients with primary recurrent and* de novo* primary MN were submitted to changes in maintenance immunosuppressive regimen, but no standard strategy was identified; 31 patients presented partial or complete remission, and glomerulopathy appeared not to impact graft and patient survival.

## 1. Introduction

Membranous nephropathy (MN) is a common cause of nephrotic syndrome, and it is a prototype of autoimmune glomerular diseases [1]. In native kidneys, MN is more common in white men, adults in the fourth or fifth decade of life, and the elderly population [2]. It can be idiopathic in 70-80% of cases or secondary to infections (hepatitis B and C), drugs, neoplasia, systemic lupus erythematosus (SLE), and others [3]. Membranous nephropathy may occur in renal allografts as a recurrent or* de novo* disease [1]. Incidence of recurrent MN ranges from 10 to 45% and its impacts on transplants outcomes are controversial [4]. Recent findings in idiopathic MN suggest that in most patients the disease is caused by PLA2R autoantibodies. Such autoantibodies are involved in 50-60% of the cases of recurrent MN. Monitoring anti-PLA2R titers during follow-up helps to predict MN recurrence, and certain immunosuppressive treatments of anti-PLA2R positive patients may prevent recurrence [5, 6]. Nevertheless, this is not a marker that reveals the development of MN in all cases. It was already reported that some patients with anti-PLA2R antibodies at the time of transplantation would not develop MN recurrence [5, 7–9].

There are scarce data about clinical presentation and outcomes of posttransplant MN, and few reports include a large number of patients. Thus, all additional information about this glomerulopathy can be useful in patient management. This study aimed to describe clinical, laboratorial, and histological characteristics, as well as treatment and outcomes of patients with posttransplant MN in a high-volume transplant center.

## 2. Methods

### 2.1. Design and Population

This retrospective cohort included adult patients with posttransplant MN transplanted between 1983 and 2015 in a single center that performs about 900 transplants per year. Only patients with histological diagnosis (optical microscopy and immunofluorescence) of MN in kidney grafts were included. Patients were identified through biopsies database and/or were selected from those followed in the Glomerulopathies Section. Protocol biopsies are not routinely performed in the center. Data were obtained from medical records and electronic databases. The study protocol was approved by the local Ethics Committee (CAAE: 24309913.1.0000.5505).

### 2.2. Definitions

Proteinuria was defined as urinary protein excretion superior to 0.3 g per day in a 24-hour urine collection or 0.3g/g in protein-to-creatinine ratio (UPr/Cr) determined in a random urine specimen [10].

Renal function was assessed by serum creatinine (Cr) or by estimated glomerular filtration rate (eGFR) using 4-variable MDRD formula [11]. The last observation carried forward (LOCF) adjustment was used for missing GFR values, attributing 10 mL/min to patients who lost the graft and the last available value for those who died or lost follow-up. Baseline serum Cr was obtained by the average of last three measurements performed before posttransplant MN diagnosis. Graft dysfunction was defined as ≥ 50% or ≥ 0.3 mg/dL increase in baseline serum Cr confirmed in two different measurements.

Partial remission was defined as Cr stabilization in a value up to 25% above the baseline level associated with decrease of proteinuria by 50% or greater and <3.0 g/g when initial values were above 3.0 g/g. Complete remission was defined as serum Cr stabilization associated with proteinuria <0.3 g/g [12]. Spontaneous remission was defined as partial or complete remission without additional immunosuppressive treatment. All renal biopsies were processed according to standard techniques for light microscopy and immunofluorescence microscopy. Electron microscopy was not performed.

The immunosuppressive therapy was individualized according to our center protocols. Induction therapy, when indicated, consisted of anti-CD25 monoclonal antibodies or antithymocyte globulin; maintenance immunosuppression was based on calcineurin inhibitor (CNI, cyclosporine or tacrolimus) plus steroid and an antiproliferative drug (mycophenolate, azathioprine, or mTOR inhibitor).

### 2.3. Statistical Analysis

Categorical variables were expressed as frequency and percentages and compared using Chi-square or Fisher's exact test. Continuous variables were presented as mean and standard deviation (SD) and median when indicated; comparison between groups was performed using the Student's* t*-test. Survival analysis was obtained using the Kaplan-Meier method. Statistical analysis was performed using IBM SPSS® 22 Statistics software, and p value was considered significant when <5%.

## 3. Results

### 3.1. Demographics

Between 1983 and 2015, 12,643 kidney transplants (KT) were performed and 41 patients with posttransplant MN were identified. Patients were predominantly male (58.5%), with a mean age of 49.4 ± 13.2 years; 36.6% presented documented MN as chronic kidney disease etiology. Most patients received kidneys from living donors (63.4%) with a mean age of 38.1 ± 16.3 years.

Most patients received no induction therapy (78.1%) and the initial maintenance immunosuppressive regimen was based on calcineurin inhibitor, steroid, and azathioprine in 56.1%, in accordance with the transplant center protocol during the study period. More detailed information about demographics is available in Table 1.

### 3.2. Clinical Presentation

Fifteen cases were considered as recurrent primary MN as patients have chronic kidney disease (CKD) due to biopsy-confirmed MN; 3 were class V lupus nephritis: 2 of this had previous diagnosis of lupus and 1 was classified as unknown etiology for CKD; 14 had defined causes for renal disease and were considered as* de novo* cases: 7 showed an underlying cause for posttransplant MN and were considered as secondary (brain, uterus, anus, thyroid, and prostate neoplasias, hepatitis C, and hepatitis B) and 7 were primary posttransplant MN; and 9 cases were considered primary but it was not possible to distinguish between* de novo* MN and recurrence, as patients had CKD for unknown or nonspecific chronic glomerulonephritis.

The most frequent initial manifestations of posttransplant MN were proteinuria (75.6%, 3.2 ± 1.2 g) and graft dysfunction (34.1%). Proteinuria > 0.3 g/day was observed at a median time of 40 months (ranging from 4 to 74) after KT. The mean levels of serum albumin and cholesterol were 3.4 ± 0.6g/dL and 198.8 ± 58.6 mg/dL, respectively. Compared to baseline values, eGFR decreased 26.8 ± 16.4% at diagnosis. Diagnostic allograft biopsy was performed 3.7 ± 2.5 months after the onset of proteinuria and a median time of 41 months (ranging from 12.3 to 87.8) after KT. MN stage 2 was the main histological presentation (60.9%), followed by stages 1 (24.4%) and 3 (14.7%) (Table 2).

### 3.3. Treatment

Patients with secondary posttransplant MN received renoprotection and treatment of underlying disease. Among patients with primary forms, 15 (45.5%) received angiotensin-converting enzyme inhibitors (ACEI) and/or angiotensin II receptor blockers (ARB). Maintenance immunosuppressive regimen was modified in 20 of the 33 patients (60.6%) with primary MN: 9 patients received high oral prednisone doses (≥0.5mg/kg/day), with or without methylprednisolone pulse therapy; 4 patients were converted from azathioprine to mycophenolate; mTOR inhibitors were withdrawn in 3 patients, 2 of which were maintained temporarily on dual immunosuppressive regimen and 1 received mycophenolate instead; in 4 patients, modified Ponticelli regimen [14] was indicated, and antiproliferative drug was withdrawn. No patient received rituximab.

### 3.4. Outcomes

Fifteen patients had an apparently spontaneous remission. There were no significant differences between patients who presented spontaneous remission and those who did not remit or only remitted after immunosuppressive therapy concerning recipient age (47.6 ± 14.7 versus 50.4 ± 12.4 years old, p=0.514); donor source (living) (60 versus 65.4%, p=0.749); time since KT at diagnosis (36.8 ± 38.7 versus 62.9 ± 56 months, p=0.118); serum creatinine (1.7 ± 0.8 versus 1.8 ± 1.5 mg/dL, p=0.835); proteinuria (3.5 ± 4.4 versus 2.9 ± 2.6 g, p=0.613); posttransplant MN category (recurrence) (48.4 versus 20%, p=0.152); or maintenance immunosuppression with mycophenolate (34.6 versus 33.3%, p=1.000).

Partial and complete remissions were observed in 25 (61%) and 11 (26.8%) patients, respectively, and occurred 358 ± 180 days after diagnosis (Table 3). Patients who presented partial or complete remissions were younger (46.8 ± 12.3 versus 57.3 ± 13.2, p=0.027), a higher proportion received living donor transplants (74.2 versus 30%, p=0.022), and a lower proportion was on mycophenolate (22.6 versus 70%, p=0.017) (Table 4).

One year after posttransplant MN diagnosis, proteinuria and serum creatinine were 2.6 ± 3 g/24h and 2.2 ± 1.1 mg/dL, respectively. Adjusting for losses and deaths, mean eGFR was 34.2 ± 18.7 mL/min (Table 3). 1-, 3-, 5-, and 10-year patient survival was 97.5%, 94.7%, 88.3%, and 88.3%, respectively. Causes of death were infection (n=2), cardiovascular event (n=1), malignancy (n=1), and unknown (n=1). Death-censored graft survival at these periods was 95.1%, 86.9%, 86.9%, and 68.4%, respectively. Five of the 13 graft losses were attributed to posttransplant MN, and the remaining 8 cases were due to unspecific interstitial fibrosis and tubular atrophy.

As expected, one year after posttransplant MN diagnosis, patients who presented partial or complete remission showed lower serum creatinine (2.0 ± 1.0 versus 2.8 ± 1.3 mg/dL, p=0.049), higher eGFR (41.8 ± 18.9 versus 27.0 ± 12.9 mL/min, p=0.028), and lower proteinuria (1.5 ± 1.6 versus 6.1 ± 3.7 g, p<0.001). During the follow-up, there was a trend for a lower rate of deaths in patients who remitted (6.5 versus 30%, p=0.083), but the rate of graft loss was similar (35.5 versus 50%, p=0.472).

## 4. Discussion

This study evaluated demography, clinical and histological presentation, treatment, and outcomes of a relatively large cohort of patients with posttransplant MN.

Unfortunately, a high proportion of patients did not have a confirmed diagnosis of chronic kidney disease (CKD) etiology (unknown and not defined chronic glomerulonephritis), which impairs the precise classification on recurrent and* de novo *cases. Besides, it is possible that some patients considered as having CKD due to hypertension actually have other underlying causes and that hypertension is secondary to renal disease.

More than a half of our cohort consisted of living donor transplants. This probably reflects the local practice in the early years of our transplant program, when living donor transplants were more common. The impact of the donor type on the risk of MN recurrence remains controversial [15].

Initial clinical and/or laboratorial presentation occurred late after KT and high proportion of patients presented nephrotic proteinuria, hypoalbuminemia, dyslipidemia, and graft dysfunction. Two patterns of MN recurrence were previously described [16]: “early recurrence” (within the first 6 months after KT), more common between living related donation, is generally oligosymptomatic, with mild proteinuria; on the other hand, “late recurrence” usually evolves with overt nephrotic syndrome. Since we did not perform protocol biopsies, even in the presence of isolated subnephrotic proteinuria, most of our cases were diagnosed in later stages. In this regard, we cannot rule out a higher rate of subclinical MN recurrence in our patients, as renal biopsies were not always performed in the absence of overt proteinuria.

Regarding specific treatment, we observed a wide variety of approaches and a lack of standardization. This was probably due to the lack of a gold standard treatment for this glomerulopathy in KT recipients, who are already on immunosuppressive therapy. More recently, good results were described with the anti-CD20 rituximab for posttransplant MN treatment and prophylaxis. However, evidence is based on small sample size noncontrolled studies. Ideal dose and safety are important aspects to be evaluated [16–18]. Noteworthy, rituximab is not available or reimbursed by Brazilian government for this off-label clinical indication.

As expected and widely reported for MN on native kidneys and allografts, about one-third of patients presented spontaneous remission [17, 19] and the majority of patients had complete or partial remission. The impact of posttransplant MN on allograft survival remains controversial [4]. In fact, in our study graft and patient survival were similar to those previously reported by our center [20–22].

Although the sample did not allow robust conclusions, we observed that remission rates were more frequent in younger recipients receiving living donor kidneys. Higher remission rates in younger patients were previously reported by studies on native kidneys [23]. However, to the best of our knowledge, there are no previous reports linking remission rates and donor source.

This study has some limitations that should be pointed out: (a) it is a single center, retrospective study, including patients transplanted in different decades; (b) it was not possible to estimate posttransplant MN incidence, since there was not a systematic recording or database for glomerular diseases in our center; (c) we did not assessed the antibodies against phospholipase A2 receptors (PLA2R) and thrombospondin type I domain-containing 7A (THSD7A), whose presence and intensity are related to recurrence; and (d) C4d was not routinely performed on graft biopsies. As strengths, this is a relatively large sample size, considering the low prevalence of the disease. Besides, the detailed description of diagnosis and treatment adds important information about the clinical management of such patients.

In summary late onset proteinuria, hypoalbuminemia, hypercholesterolemia, and graft dysfunction were the main clinical manifestations of posttransplant MN. RAAS blockade was common and there was not a standard treatment. The course was benign, with high proportion of remission rates and no impact on survival.

## Figures and Tables

**Table 1:** Demographic characteristics of patients with posttransplant MN.

Variables	N = 41
Recipient gender – male, n(%)	24 (58.5)
Recipient age (years), mean ± DP	49.4 ± 13.2
Pretransplant dialysis, n(%)	40 (97.5)
Time on dialysis (months), mean ± DP	22.6 ± 24.1 (median = 16)
Etiology of end-stage renal disease, n(%)	
*Membranous nephropathy*	15 (36.6)
*Lupus nephritis*	2 (4.9)
*Unknown*	9 (22)
*Chronic glomerulonephritis*	5 (12.2)
*Hypertension*	5 (12.2)
*Diabetes*	2 (4.9)
*Chronic pyelonephritis*	1 (2.4)
*Urological *	1 (2.4)
*Cortical necrosis*	1 (2.4)
Panel reactive antibodies (%), mean ± DP	7.85 ± 22.6 (median = 0)
Donor source, n(%)	
*Living*	26 (63.4)
*HLA identical*	6 (14.6)
*HLA haploidentical*	16 (39)
*HLA distinct*	4 (9.8)
*Deceased*	15 (36.6)
*Standard criteria donor*	10 (24.4)
*Expanded criteria donor*^*1*^	5 (12.2)
Donor age (years), mean ± DP	38.1 ± 16.3
HLA mismatches, mean ± DP	2.3 ± 1.8
Cold ischemia time (hours), mean ± DP	25 ± 9
Induction therapy, n(%)	
*None*	32 (78.1)
*Basiliximab*	1 (2.4)
*Thymoglobulin*	8 (19.5)
Initial immunosuppressive regimen, n(%)	
*CNI-PRED-AZA*	23 (56.1)
*CNI-PRED-MPA*	14 (34.1)
*CNI PRED-SRL*	4 (9.8)

PRED: prednisone; CNI: calcineurin inhibitor; AZA: azathioprine; MPA: mycophenolic acid; SRL: sirolimus; HLA: human leukocyte antigens.

^1^United Network for Organ Sharing (UNOS) criteria [13].

**Table 2:** Clinical presentation of patients with posttransplant MN.

**Variables**	**N = 41**
Proteinuria > 0.3 g/g or g/24h, n(%)	31 (75.6)
Proteinuria^1^ (g/g or g/24h), mean ± DP	3.2 ± 1.2
Time to onset of proteinuria > 0.3 g/g or g/24h (months), mean ± DP	49.5 ± 49.7 (median = 40)
Serum albumin^1^ (g/dL), mean ± DP	3.4 ± 0.6
Serum total cholesterol^1^ (mg/dL), mean ± DP	198.8 ± 58.6
Serum triglycerides^1^ (mg/dL), mean ± DP	178.5 ± 78.7
Systolic blood pressure^1^ (mmHg), mean ± DP	131 ± 7.8
Diastolic blood pressure^1^ (mmHg), mean ± DP	82.2 ± 6.5
Graft dysfunction^1^, n(%)	14 (34.1)
Serum creatinine^1^ (mg/dL), mean ± DP	1.7 ± 0.6
eGFR^1^ (mL/min/1.73m^2^), mean ± DP	41.9 ± 14.3
eGFR decrease^1^ (%), mean ± DP	26.8 ± 16.4
Time between proteinuria and graft biopsy (months), mean ± DP	3.7 ± 2.5
Time between KT and graft biopsy (months), mean ± DP	53.4 ± 51.5 (median = 41)
Histological stages of MN, n(%)	
* Stage 1*	10 (24.4)
* Stage 2*	25 (60.9)
* Stage 3*	6 (14.7)

eGFR: estimated glomerular filtration rate; KT: kidney transplant.

^1^ At diagnosis.

**Table 3:** Posttransplant MN outcomes.

**Variables**	**N = 41**
Time on follow-up (months), mean ± DP	108.2 ± 52.8
Partial remission, n(%)	20 (48.78)
Complete remission, n(%)	11 (26.8)
Spontaneous remission, n(%)	15 (36.6)
Time to remission since diagnosis (days), mean ± DP	358 ± 180
Proteinuria (g/g or g/24h)^1^, mean ± DP	2.6 ± 3 (median = 2)
Serum creatinine (mg/dL)^1^, mean ± DP	2.2 ± 1.1
eGFR (mL/min/1.73m^2^)^1^, mean ± DP^ 1^	38.2 ± 19.5
eGFR - LOCF (mL/min/1.73m^2^)^1^, mean ± DP	34.2 ± 18.7

eGFR: estimated glomerular filtration rate; LOCF: last observation carried forward.

^1^ 1 year after diagnosis.

**Table 4:** Risk factors for posttransplant MN remission.

**Variables**	**Partial or complete remission** **N=31**	**No remission** **N=10**	**p value**
Recipient age (years old), mean ± DP	46.8 ± 12.3	57.3 ± 13.2	0.027
Time after KT at diagnosis (months), mean ± DP	54.6 ± 53.7	49.4 ± 46.4	0.782
Serum creatinine at diagnosis (mg/dL), mean ± DP	1.6 ± 0.7	2.1 ± 2.3	0.351
Proteinuria at diagnosis (g/24h or g/g), mean ± DP	2.9 ± 3.2	4.1 ± 3.5	0.337
Living donor, n(%)	23 (74.2)	3 (30)	0.022
RAAS blockade, n(%)	26 (83)	9 (90)	1.000
Change in maintenance IS after diagnosis, n(%)	16 (51)	4 (40)	0.719
Treatment with high dose steroids, n(%)	14 (45.2)	3 (30)	0.480
Treatment with Ponticelli scheme, n(%)	1 (3.2)	2 (20)	0.142

KT: kidney transplant; RAAS: *renin angiotensin aldosterone system.*
